# Quality of Medicines Commonly Used in the Treatment of Soil Transmitted Helminths and Giardia in Ethiopia: A Nationwide Survey

**DOI:** 10.1371/journal.pntd.0003345

**Published:** 2014-12-04

**Authors:** Sultan Suleman, Gemechu Zeleke, Habtewold Deti, Zeleke Mekonnen, Luc Duchateau, Bruno Levecke, Jozef Vercruysse, Matthias D'Hondt, Evelien Wynendaele, Bart De Spiegeleer

**Affiliations:** 1 School of Pharmacy, Jimma University, Jimma, Ethiopia; 2 Drug Quality and Registration (DruQuaR) Group, Faculty of Pharmaceutical Sciences, Ghent University, Ghent, Belgium; 3 School of Medical Laboratory Sciences, Jimma University, Jimma, Ethiopia; 4 Department of Virology, Parasitology and Immunology, Faculty of Veterinary Medicine, Ghent University, Merelbeke, Belgium; 5 Department of Comparative Physiology and Biometrics, Faculty of Veterinary Medicine, Ghent University, Merelbeke, Belgium; Swiss Tropical and Public Health Institute, Switzerland

## Abstract

**Background:**

The presence of poor quality medicines in the market is a global threat on public health, especially in developing countries. Therefore, we assessed the quality of two commonly used anthelminthic drugs [mebendazole (MEB) and albendazole (ALB)] and one antiprotozoal drug [tinidazole (TNZ)] in Ethiopia.

**Methods/Principal Findings:**

A multilevel stratified random sampling, with as strata the different levels of supply chain system in Ethiopia, geographic areas and government/privately owned medicines outlets, was used to collect the drug samples using mystery shoppers. The three drugs (106 samples) were collected from 38 drug outlets (government/privately owned) in 7 major cities in Ethiopia between January and March 2012. All samples underwent visual and physical inspection for labeling and packaging before physico-chemical quality testing and evaluated based on individual monographs in Pharmacopoeias for identification, assay/content, dosage uniformity, dissolution, disintegration and friability. In addition, quality risk was analyzed using failure mode effect analysis (FMEA) and a risk priority number (RPN) was assigned to each quality attribute. A clinically rationalized desirability function was applied in quantification of the overall quality of each medicine. Overall, 45.3% (48/106) of the tested samples were substandard, i.e. not meeting the pharmacopoeial quality specifications claimed by their manufacturers. Assay was the quality attribute most often out-of-specification, with 29.2% (31/106) failure of the total samples. The highest failure was observed for MEB (19/42, 45.2%), followed by TNZ (10/39, 25.6%) and ALB (2/25, 8.0%). The risk analysis showed that assay (RPN = 512) is the most critical quality attribute, followed by dissolution (RPN = 336). Based on Derringer's desirability function, samples were classified into excellent (14/106,13%), good (24/106, 23%), acceptable (38/106, 36%%), low (29/106, 27%) and bad (1/106,1%) quality.

**Conclusions/Significance:**

This study evidenced that there is a relatively high prevalence of poor quality MEB, ALB and TNZ in Ethiopia: up to 45% if pharmacopoeial acceptance criteria are used in the traditional, dichotomous approach, and 28% if the new risk-based desirability approach was applied. The study identified assay as the most critical quality attributes. The country of origin was the most significant factor determining poor quality status of the investigated medicines in Ethiopia.

## Author Summary

Access to medicines of good quality improves the chances of successful treatment for individual patients and promotes better outcomes for public health in general. At present, the prevailing strategy for improving access to medicines for neglected tropical diseases (NTDs) is drug donation programs. However, the presence of poor quality medicines in the market is a global threat on public health, especially in developing countries by critically risking efforts of treatment and control of diseases in general and the NTDs in particular. Conventionally, medicine quality has been ignored in NTDs, though scattered reports show that serious problems exist. Therefore, we assessed the quality of two commonly used anthelminthic drugs (MEB and ALB) and one antiprotozoal drug (TNZ) in Ethiopia. The analytical results were converted into conclusions using two systems: the traditional dichotomous pharmacopoeial specification-compliance based approach and the risk-based Taguchi quantitative desirability approach. Overall, the results showed high prevalence of poor quality of the three medicines, mainly determined by the country of origin. We conclude that risk-based regulatory quality control procedures should be based on identification of the most critical quality attribute and apply desirability functions to quantify and classify the quality of medicines.

## Introduction

Intestinal parasites are a diverse group of organisms that include single-celled protozoans and multi-cellular intestinal helminths that affect the gastro-intestinal tract of humans and other animals [1]. Soil-transmitted helminthiasis is caused primarily by four species of nematodes, i.e. *Ascaris lumbricoides* (roundworm), *Trichuris trichiura* (whipworm), and *Ancylostoma duodenale* and *Necator americanus* (hookworms) that parasitize human gastrointestinal tract [2]. These major human soil-transmitted helminths (STH) have significant impact on human health in many parts of the world, particularly in developing countries [3]. If not treated early and efficacious, they may lead to malnutrition, chronic diarrhea, anemia, and other public health problems that can impair physical and intellectual development in children [4]–[6].

Currently, four drugs are recommended by the World Health Organization (WHO) for STH: MEB, ALB, levamisole and pyrantel pamoate [7], [8]. MEB and ALB are increasingly deployed in mass drug administration programs [8] which require a single drug administration to all subjects without prior diagnosis or checking for contra-indications. For this reason, the two benzimidazole 2-carbamates MEB and ALB (chemical structures presented in S1 Supporting information) are preferred over levamisole and pyrantel pamoate, which require weight-based dosing and which are also intrinsically less potent.

Literature reports indicate that TNZ, a 5-nitroimidazole compound (S1-1 Supporting information), also has some anthelmintic efficacy [9], although it is therapeutically mainly used against protozoan infections and infections caused by anaerobic bacteria in humans. As such TNZ is often used by the same patients treated with STH drugs [10], [11].

Effective treatment and prevention strategies for these neglected tropical diseases can be delivered cheaply, but reports of treatment failure are frequent in developing countries most likely because of poor quality medicines, which includes spurious/falsely labeled/falsified/counterfeit (SFFC) medicines, chemical and/or physicochemical instability, inappropriate storage and transport, and poor quality control during manufacturing and importing medicines [12]. SFFC medicines are medicines that are deliberately and fraudulently mislabeled with respect to identity and/or source and include products with the correct ingredients or with wrong ingredients, without active ingredients, with insufficient or too much active ingredient, or with fake packaging [13]. Substandard medicines, i.e. not having the appropriate quality (which is expected to be equivalent to the regulatory quality), may be SFFC but also approved medicines. In a quality survey in Nigeria, 48% of the samples of different categories of medicines were found to be outside the British Pharmacopoeia (BP) limits for active pharmaceutical ingredient (API) assay. Some medicines were even lacking the active ingredient [14]. The use of substandard medicines may result in therapeutic failure, resistance development, and occurrence of serious adverse events or even death due to excessive dose or the presence of toxic impurities [15]–[17]. A study conducted in sub-Saharan Africa in 2010 on the quality of selected anti-malarial medicines reported 64% overall quality failure in Nigeria, from which one artemisinin-based anti-malarial drug sample did not contain any of artemether API [18].

The presence of substandard and SFFC medicines not only poses threats to the individual users in terms of the health and side effects experienced, but also to the public and government in terms of trade relations and economic implications [19]. Hence, like many other public health problems, the issue of the presence of these substandard and SFFC medicines for public consumption should receive careful attention in developing countries [16].

Finished pharmaceutical products (FPPs) are tested for quality by assessing whether they meet pharmacopoeial or any other approved specifications. If not, they are discarded as non-conforming. This is a dichotomous decision without differentiation of the seriousness of failure and/or importance of quality attributes towards clinical use for the patient [20]. The evaluation of quality of any product poses thus a common problem due to a multiplicity of measures which must be balanced one against the other. Even when the quality attributes are precisely measurable, a serious challenge exists in combining the individual measurements into one index representing the total quality [21]. Such balance problems can be solved by using a Derringer's desirability function [22].

In general, this study was carried out to assess the pharmacopoeial quality of three medicines (MEB, ALB and TNZ) circulating in Ethiopia. The quality in terms of quality attributes like assay/content, dosage uniformity, dissolution, disintegration and friability was evaluated. The criticality of the quality attributes was assessed using FMEA risk-based analysis and Derringer's desirability function was applied to obtain one global quality index for each sample investigated.

## Methods

### Materials

MEB USP working standard [Cadila Pharmaceuticals (Ethiopia)], ALB reference standard [Greenfield Pharmaceuticals (China)] and TNZ reference standard [Greenfield Pharmaceuticals (China)] were kindly donated from Food, Medicine and Health-care Administration and Control Authority (FMHACA) of Ethiopia and used as received. Purified ultra pure water was obtained by water purification system (Thermofischer Scientific, USA, 18.2 MΩ.cm at 25°C). All other chemicals used in this study were analytical grade and used as received.

### Sample collection

The sampling strategy was defined following the Medicine Quality Assessment Reporting Guidelines (MEDQUARG) as proposed by Newton PN et al., 2009 [23] based on the questions: “Are there medicines of poor quality in the formal distribution outlets in Ethiopia? If there are, what is the prevalence of these poor quality medicines?” Moreover, since there is a possible influence of origin and distribution conditions on medicines quality as received by the patient, we included the different formal outlets that are in practice used by patients in Ethiopia. So, we also looked at the following question: “Is there a difference in quality of medicines (1) among the different levels of medicines outlets? (2) across different geographic areas of the country? (3) among the two national economies: government and privately owned medicines outlets and (4) among the different countries of origin”. Therefore, in function of the questions, sampling units were defined to be the medicines sold from the drug retail outlets of the formal supply chain in the country, the different levels of the supply chain system in Ethiopia (drug stores incl. health centers, pharmacies incl. hospital pharmacies, wholesalers), the geographic areas, government/privately owned medicines outlets and country of origin.

Based on the sampling strategy, 106 drug samples were collected between January and March 2012 through multilevel stratified random sampling from all the levels of the supply chain system of the country (n = 3) covering all types of government and privately owned drug outlets (n = 2). All available drug samples of the three study medicines were collected from each of the selected drug outlet. Through proportional allocation to each stratum of the supply chain, 59.4% (n = 63) of the drug samples were collected from drug stores; 36.8% (n = 39) were from pharmacies while the remaining 3.8% (n = 4) samples were from wholesales. 17.9% (n = 7) of pharmacy collected drug samples were obtained from hospitals, while four of the drug samples collected from drug stores was from health centers. Depending on the geographic locations and drug markets, the samples were collected from 7 major cities of the country: Addis Ababa, Hawasa (and its region including Arbaminch and Shashemene), Jimma, Assosa (and its region including Nekemte), Adama, Mekele and Bahirdar; which represent all four directions starting from Addis Ababa, the major central commercial center. All samples were tablet formulations and purchased anonymously by mystery shoppers from local area who were trained before. The mystery shoppers stated, if needed, that they were a travelling five member family and the family head, a man of 35 years old, abruptly caught a stomach ache (‘kurtet’ in Amharic) due to worm infestations and requested the dispenser at the medicine outlet for some mebendazole (for ‘kurtet’) and albendazole tablets (for ascariasis) as he used both medicines from his past experiences. At the same time, the family's 18 years old son was suffering from diarrhea and thus requested the dispenser for any medicines which could be given for him describing that he was taking tinidazole tablets two months ago for similar symptoms. Since the travelling family was in a worry of coming up with shortage of the medicines while travelling they requested a sufficient quantity of tablets of the medicines.

The mystery shoppers were blinded about the purpose of the study and only instructed to purchase medicines in their original primary packaging as supplied by the manufacturer. For the purpose of this study, the relevant information of all collected samples was recorded on a standard form as soon as leaving the drug outlet and entered into database. The information included the level of the drug outlet, place/city of collection, name of the active pharmaceutical ingredient, the country of origin, manufacturing company, expiry date, manufacturing date, batch/lot number, and labeled dose (strength) of the active ingredient. Medicines purchased from a specific outlet, labeled with a specific generic name or brand name, strength, number of units per strip/package, batch number, country of origin, manufacturing and expiry dates were considered as one sample. Since the mystery shoppers stated that they were a travelling five member family, they were able to buy enough units per sample. For MEB, 50 tablets per sample were purchased while for ALB and TNZ, a sample contained 100 tablets. The samples were stored at ambient temperature (20°C to 25°C) until tested, with a storage period of maximally 3 months before testing, and none of samples had expired at the time of testing.

### Test methods for products quality

The quality control laboratory tests were performed in Jimma University Laboratory of Drug Quality (JuLaDQ), Jimma, Ethiopia. JuLaDQ follows a quality system extended from its collaborating laboratory, Laboratory of Drug Quality and Registration (DruQuaR) of Ghent University, 9000-Ghent, Belgium.

The laboratory tests were carried out according to the general and individual monographs specified in different Pharmacopoeias, as indicated in S1-2 supporting information.

Instrument performance and system suitability tests were successfully performed for the analytical instruments and HPLC methods, respectively.

For any drug product, identification of the active pharmaceutical ingredient (API) is a critical quality attribute. The three drugs (ALB, MEB and TNZ) belong to biopharmaceutical classification system (BCS) class II, with low aqueous solubility and high permeability [24], [25]. Moreover, disintegration is an integral part of and/or pre-requisite for dissolution of immediate release dosage forms [26].

Therefore, quality attributes based upon which the products were evaluated were defined to be identification, assay/content, dissolution, dosage uniformity, disintegration and friability tests. Quality failure was defined as a sample failing any single test of the aforementioned tests for which it was evaluated.

Details of the laboratory test methods used to evaluate the study medicines are presented in S1-3 supporting information.

### Risk analysis

Risk analysis is a general quality tool which has its roots in engineering [27], but is now becoming a well-established tool in the pharmaceutical field as well. As such, ICH has devoted a separate guideline (Q9) to quality risk management, which is being embraced by pharmaceutical authorities [28]. Risk analysis, i.e. the estimation of the risk associated with the identified hazards, is an important part of this global risk management. Several quality risk management tools like FMEA (Failure Mode Effects Analysis) are available, as mentioned by ICH in Q9. Therefore, FMEA was used to evaluate the criticality of product quality attributes in this study. Criticality was evaluated using RPN, based on evaluations about the probability of occurrence of the failure (*O*), the severity of the failure (*S*) and the probability of not detecting the failure (*D*). These judgments are converted into numerical values using descriptive scales and finally combined in the RPN [29] by means of Equation (1):
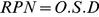
(1)


Used scales for severity, occurrence and detectability of failure are presented in Tables 1 to 3 [30]. For severity ratings, five pharmaceutical experts in Belgium (4) and Ethiopia (1) (S1-4 supporting information) were assigned to score it and the median score was taken. For occurrence, literature was reviewed for the three products (MEB, ALB and TNZ) in Africa and for other drugs in Ethiopia as there was no previous quality study conducted for these three products in Ethiopia. In Nigeria, 48% of MEB samples contained amounts of active ingredient outside the appropriate assay limits [31]. Assay based pharmaceutical quality assessment in Kenya reported very poor quality for majority of marketed anthelmintic preparations [32]. Therefore, the highest occurrence score of 8 was assigned for assay. Studies conducted in Ethiopia indicated that the occurrence of failure of identification, disintegration and friability tests are very low making the scores assigned to each of these failures to be 1 [18], [33], [34]. Since 19.1% (8/42) of our MEB samples did not meet the pharmacopoeial acceptance criteria for dosage form uniformity, the probability of occurrence of this failure is moderately high and thus a score of 6 was assigned for its occurrence. For scoring the detectability, the scaling ranged from the low score assigned to the easiest detection to the highest score for the more difficult detection method. Friability can be detected through simple visual/weighing observation; hence, a score of 1 was assigned to its detectability. On the other hand, assay and dissolution studies involve quantitative tests, requiring fully equipped laboratory system and trained personnel. Therefore, detectability was scored to be 8 for each of these failure modes. Since identification requires field tests like color reactions and/or TLC, a score of 5 was assigned to detectability of identity failures.

**Table 1:** Evaluation criteria and ranking system for the severity of effects.

*Effect*	*Criteria: severity of effect*	*Rank*
Hazardous	Failure is hazardous, and occurs without warning. It suspends operation of the system	10
Serious	Failure involves hazardous outcomes and/or noncompliance with government regulations or standards	9
Extreme	Product is inoperable with loss of primary function. The system is inoperable	8
Major	Product performance is severely affected but functions. The system may not operate	7
Significant	Product performance is degraded. Comfort or convince functions may not operate	6
Moderate	Moderate effect on product performance. The product requires repair	5
Low	Small effect on product performance. The product does not require repair	4
Minor	Minor effect on product or system performance	3
Very minor	Very minor effect on product or system performance	2
None	No effect	1

**Table 2:** Evaluation criteria and ranking system for the occurrence of failure.

*Probability of failure*	*Possible failure rates*	*Rank*
Extremely high: failure almost inevitable	≧1 in 2	10
Very high	1 in 3	9
Repeated failures	1 in 8	8
High	1 in 20	7
Moderately high	1 in 80	6
Moderate	1 in 400	5
Relatively low	1 in 2000	4
Low	1 in 15,000	3
Remote	1 in 150,000	2
Nearly impossible	≦1 in 1,500,000	1

**Table 3:** Evaluation criteria and ranking system for the detection of a cause of failure.

*Detection*	*Criteria: likelihood of detection by design control*	*Rank*
Absolute uncertainty	Design control does not detect a potential cause of failure or subsequent failure mode; or there is no design control	10
Very remote	Very remote chance the design control will detect a potential cause of failure or subsequent failure mode	9
Remote	Remote chance the design control will detect a potential cause of failure or subsequent failure mode	8
Very low	Very low chance the design control will detect a potential cause of failure or subsequent failure mode	7
Low	Low chance the design control will detect a potential cause of failure or subsequent failure mode	6
Moderate	Moderate chance the design control will detect a potential cause of failure or subsequent failure mode	5
Moderately high	Moderately high chance the design control will detect a potential cause of failure or subsequent failure mode	4
High	High chance the design control will detect a potential cause of failure or subsequent failure mode	3
Very high	Very high chance the design control will detect a potential cause of failure or subsequent failure mode	2
Almost certain	Design control will almost certainly detect a potential cause of failure or subsequent failure mode	1

### Desirability function

Desirability function, just like risk analysis, is a quality tool first proposed by Harrington in 1965 for use in the optimization of quality of manufactured products. The approach has basic foundation in engineering [35], [36] and is widely adopted in the manufacturing industry.

The central idea of a desirability function is to create one ball-mark figure. which is a composite number reflecting different response. This is done by mapping the value of each property/response onto a unit-less score in the range from zero to one based on the appropriateness (or desirability) of the property/response. Therefore, Derringer's desirability function was applied for the assessment of the quality of the three pharmaceutical products (MEB, ALB and TNZ). The desirability function can be used to combine multiple responses into one response called the “*overall desirability function*” D, ranging between a value of 0 (one or more product characteristics are completely unacceptable) to 1 (all product characteristics are on target). This overall desirability function D is obtained from the geometric mean of the individual desirabilities (d_i_) which provide a way to assess the quality of one property. The formula to calculate the overall D-value is presented in Equation 2: 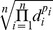
(2)


In this equation, *p_i_* was the weight or relative importance assigned to the response. For this study, *n* equals 4 since four characteristics were considered in the global evaluation of ALB and TNZ, while n  = 3 for MEB since dissolution study was not performed. The advantage of calculating the geometric mean is that when one of the criteria has an unacceptable value, the overall product will be unacceptable as well. The highest global desirability value represents the product with the highest quality.

Individual desirability functions were defined for each of the quality attributes, based on a psychophysical scale and the results obtained from the FMEA quality assessment. Desirability function possessing values in the range (0–1) classifies the conversion of the quantity value of a specific quality indicator into the assessment of the desirability (preference) of a certain condition of evaluated subject (pharmacopoeial quality of the three medicines). Among the specific ways to implement the desirability function for the corresponding estimation, a psychophysical scale of Harrington is chosen providing universal application. The scale served to establish the correspondence between physical and psychological parameters. All the numeric desirability values (0–1) of the measured parameters/quality attributes are regarded as physical parameters, while a purely subjective assessment of a researcher (e.g. excellent, good, acceptable, low, bad) to express degree of satisfaction are regarded as psychological parameters.

A rough estimation constructs a five–interval quality scale (Table 4) [37]. For assay and dissolution, a two-sided desirability function was used where it becomes zero at the lowest and upper limit. For identity and dosage form uniformity, a one-sided desirability function was used. Absence of API is assumed to be clinically completely undesirable and thus this point was assigned d = 0 where as 100%lc was assigned d = 1 (i.e. optimal desirability). Since the pharmacopoeial specification for assay is 90–110%lc for all the three products and the pychophysical Harrington's scale of quality specifies desirability range from about 0.7 to 1.0 to be good, d = 0.7 was assigned for assay values of 90 and 110%lc. Moreover, d = 0.3 was assigned for both 70% and 130%lc, while d = 0.01 was assigned to 50% and 150%lc. The individual desirability function for assay was then defined as different linear sections of different slopes in the range of 100%lc to 90%lc (slope  = 0.03), 90%lc to 70%lc (slope  = 0.02) and from 70%lc to 50%lc (slope  = 0.01). Similar but negative slopes were used for assay values greater than 100%lc, mirroring the under-dosing profile.

**Table 4:** Modified psychophysical Harrington's scale of quality and results of risk-based desirability function approach.

#	*Intervals in global desirability (D-global)*	*Quality, descriptive evaluation*	*Number of products in each quality scale (percentage)*
1	0.90–1.00	Excellent	14 (13%)
2	0.80–0.90	Good	24 (23%)
3	0.70–0.80	Acceptable	38 (36%)
4	0.37–0.70	Low*	29 (27%)
5	0.00–0.37	Bad*	1 (1%)

*Unacceptable qualities.

For dissolution, %drug release was considered. According to USP acceptance criteria (S1-2 supporting information), ALB should release 80% within 30 minutes, while TNZ should release 75% within 120 minutes. However, BP sets acceptance criteria for both drugs at 70%. Therefore, d = 1 was assigned for 100% drug release, while d = 0.7 was assigned for the average 75% and 125% drug release for both ALB and TNZ. Moreover, d = 0.3 was assigned for both 50% and 150% drug release, while d = 0.01 was assigned to 40% and 160% drug release.

For dosage uniformity, the relative standard deviation (RSD) was considered as response. According to Ph. Eur. (2012), RSD should be not more than 2%; and thus d = 1 was assigned for RSD = 0% while d = 0.7 for RSD = 2%. Following Harrington's scale, d = 0.3 was assigned for RSD of 6% and d = 0.01 for RSD of 15%; while for RSD = 25%, d was assigned to be 0.

For identity, d = 1.0 was assigned for those complying with pharmacopoeial specifications for identity and d = 0 for those which do not comply.

### Data analysis

Data entry and analysis was carried out using Statistical Package for Social Sciences software (version 16.0 for windows; SPSS). The assay was carried out in triplicate and data were expressed as mean values. The Fisher exact test was used to test the association of the binary quality attributes with the country of origin (5 origins), collection sites (7 cities) and drug outlets (3 types). A more detailed statistical data analysis, based on the fixed effects model with different response variables (product quality attributes) and different categorical covariates derived from our sampling strategy questions was done. FMEA was used to assess the criticality of the quality risks associated with each quality attribute and Derringer's desirability function was applied to evaluate quality of the products.

## Results

### Quality of the investigated products

A total of one hundred and six samples of MEB, ALB and TNZ were collected between January and March 2012 in seven major cities that represent most parts of the country considering pharmaceutical market and geographic areas. The samples had been collected from 38 premises (wholesales, pharmacies and drug stores). Of these, 42 samples were MBZ, 25 samples were ALB and 39 were TNZ samples. The origin (place of manufacturing) of samples was domestic and foreign (China, India, Korea, and Cyprus). Domestic products constituted 45.3% (48/106), followed by Indian products with 26.5% (28/106). All samples had the intended active ingredient as demonstrated by the positive identification tests. No gross mislabeling (incorrect, inadequate or incomplete identification) was observed for the samples. However, the quantitative laboratory experiments indicated that 45.3% (48/106) of the samples did not meet the expected pharmacopoeial quality specifications: 45.2% (19/42) MEB, 48.0% (12/25) ALB and 43.6% (17/39) TNZ samples. The results of the different quality control tests of the samples are presented in Table 5 and are detailed below.

**Table 5:** Pharmacopoeial quality test results by product.

*Product and strength (mg)*	*Samples failing quality parameters test*				
	*Assay*	*Dissolution*	*Dosage uniformity*	*Friability*	*Overall*
ALB (400)	8% (2/25)	42% (8/19)	0% (0/25)	20% (5/25)	48% (12/25)
MEB (100)	45% (19/42)	-	19% (8/42)	7% (3/42)	45% (19/42)
TNZ (500)	26% (10/39)	18% (7/39)	0% (0/39)	8% (3/39)	44% (17/39)
***Overall***	***29% (31/106)***	***26% (15/58)***	***7% (8/106)***	***10% (11/106)***	***45% (48/106)***

- Not performed.

#### Assay

**Figure 1:**
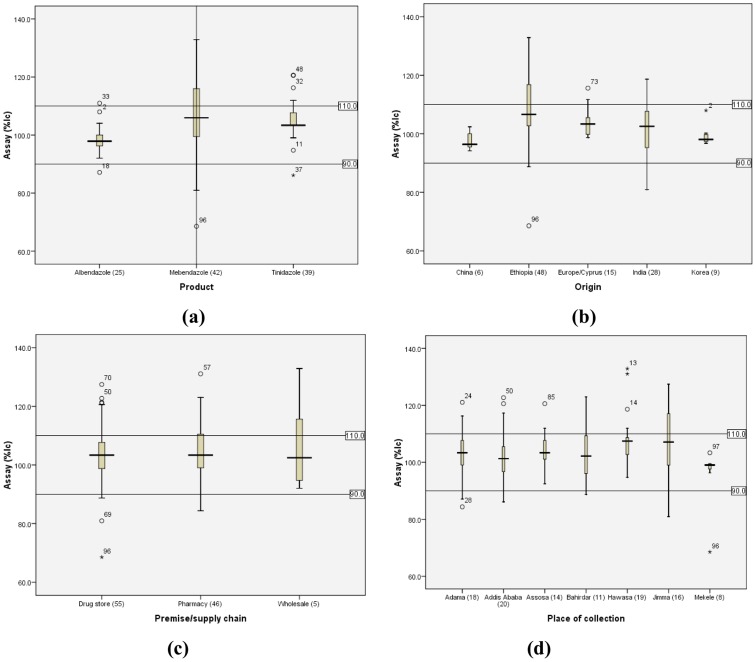
Box plot for assay versus (a) product type, (b) origin, (c) premise/supply chain and (d) place of collection. Numbers are given between the brackets.

The assay values for MEB drug products ranged from 68.6 to 132.9%lc (mean: 106.2%), while that of ALB ranged from 87.1 to 111.0%lc (mean: 98.6%). For TNZ drug products, the assay values ranged from 86.1 to 120.6%lc (mean: 105.7%). A box plot of assay by product type, country of origin, supply chain and place of collection is indicated in Fig. 1 and assay test results by product brand is presented in Table 6.

**Table 6:** Assay test results by product type.

*Drug products (n)*	*Brands (n)*	*Minimum*	*Maximum*	*Mean*	*SD*	*Median*
**MEB (42)**	M1 (14)	98.6%	131.1%	108.6%	9.7%	106.6%
	M2 (14)	68.6%	132.9%	107.6%	17.2%	107.7%
	M3 (7)	81.0%	109.4%	95.0%	10.3%	93.0%
	M4 (5)	98.7%	115.6%	105.8%	7.4%	102.2%
	M5 (1)	121.1%	121.1%	121.1%	NA	121.1%
	M6 (1)	118.7%	118.7%	118.7%	NA	118.7%
	**Sub total**	**68.6%**	**132.9%**	**106.2%**	**13.3%**	**106.0%**
**ALB (25)**	A1 (1)	92.0%	92.0%	92.0%	NA	92.0%
	A2 (9)	96.7%	108.0%	99.2%	3.5%	97.9%
	A3* (6)	94.2%	102.4%	97.5%	3.1%	96.4%
	A4 (2)	99.5%	103.3%	101.4%	2.7%	101.4%
	A5 (1)	104.0%	104.0%	104.0%	NA	104.0%
	A6 (3)	87.1%	100.0%	95.3%	7.1%	98.8%
	A7 (1)	96.3%	96.3%	96.3%	NA	96.3%
	A8 (1)	94.3%	94.3%	94.3%	NA	94.3%
	A9 (1)	111.0%	111.0%	111.0%	NA	111.0%
	**Sub total**	**87.1%**	**111.0%**	**98.6%**	**4.8%**	**97.9%**
**TNZ (39)**	T1 (3)	86.1%	99.1%	94.7%	7.5%	99.1%
	T2 (7)	107.7%	112.0%	108.9%	2.1%	107.7%
	T3* (5)	99.1%	112.0%	104.2%	4.7%	103.4%
	T4* (9)	99.1%	107.7%	102.4%	2.9%	103.4%
	T5 (8)	99.1%	120.6%	113.6%	7.6%	114.1%
	T6 (5)	94.8%	103.4%	101.6%	3.9%	103.4%
	T7* (2)	107.7%	107.7%	107.7%	0.0%	107.7%
	**Sub total**	**86.1%**	**120.6%**	**105.7%**	**7.0%**	**103.4%**
**Total (106)**		**68.6%**	**132.9%**	**104.2%**	**10.1%**	**103.4%**

*Generic products; SD =  Standard deviation; NA =  Not applicable.

This study revealed that 29.2% (31/106) of samples did not meet the pharmacopoeial acceptance specification for the assay, and thus are formally classified as substandard medicines [13]. A high failure rate, 45.2% (19/42) was found for MEB tablets followed by TNZ with failure rate of 25.6% (10/39) and 8.0% (2/25) of ALB samples. From those 31 samples failing to meet the official specification limit for assay, 80.7% (25/31) of the samples were over-dosed and 19.4% (6/31) were under-dosed. MEB samples showed the highest variation for assay test with a relative standard deviation (RSD) of 12.5%, followed by TNZ and ALB with RSD 6.7% and 4.8% of the labeled amount, respectively. Considering the time left to expiry date, all ALB samples expired in 2013, while for MEB and TNZ, the expiry date was longer, i.e. 2015/16, which can explain the difference in assay values between the 3 drug product classes.

The assay results reveal that the majority of the failed samples contain too much active ingredient that may be introduced intentionally during production (i.e., overages applied). However, as a general principle, use of an over-dose of a drug substance to compensate for loss during manufacture or degradation during a product's shelf life to extend its shelf life, is discouraged [38].

#### Disintegration test

In this study, all tablet samples met the official requirement for disintegration time test.

#### Dissolution test

**Figure 2:**
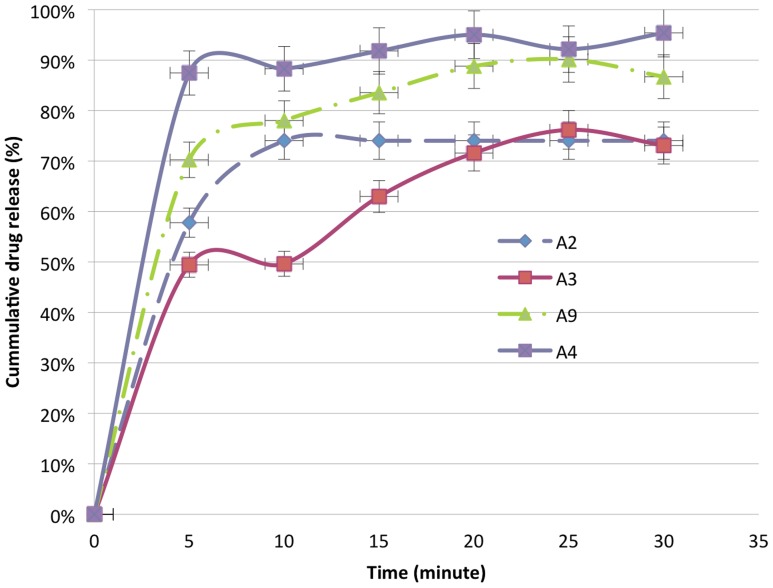
Comparative *in-vitro* release profile studies of four different products of albendazole (ALB) tablets. All data points presented are mean values of triplicate experiments (n = 3) and error bars indicate standard deviations. Percent drug release should be between 70 and 130% within 30 min.

**Figure 3:**
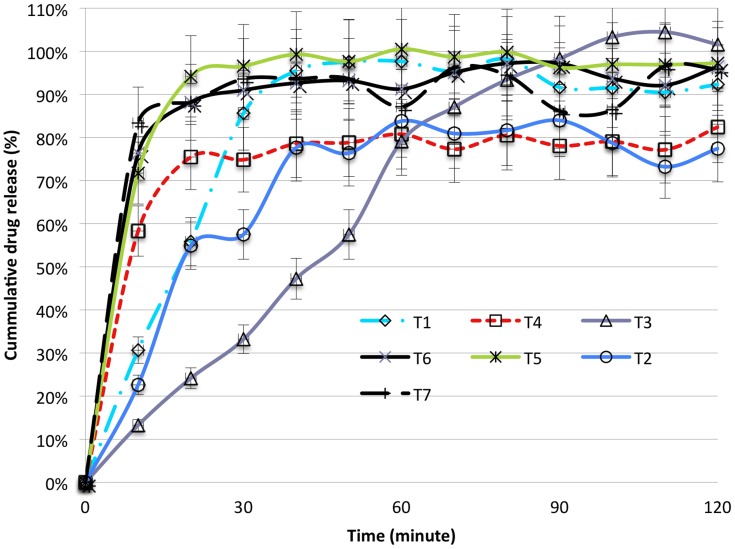
Comparative *in-vitro* release profile studies of different products of tinidazole (TNZ) tablets. All data points presented are mean values of triplicate experiments (n = 3) and error bars indicate standard deviations. Percent drug release should be between 70 and 130% within 120 min.

As shown in Table 5, from 19 ALB and 39 TNZ samples tested for their *in-vitro* dissolution, 42.1% (8/19) of ALB and 17.9% (7/39) of TNZ samples failed to meet the official tolerance limits. There is a significant difference between countries of origin with respect to the *in-vitro* dissolution profile, with all samples manufactured in Ethiopia (19/19) meeting the official tolerance limit and 25.9% (15/58) failure rate observed for the imported products. From the 11 products (4 ALB and 7 TNZ) purposefully selected for the release profile study, all three ALB brands released more than 80.0%lc in 30 minutes except one ALB generic product but a fast release was revealed from one product in which 74.0%lc was released within 10 minutes as presented in Fig. 2. All four TNZ brands and three generic products released more than 75.0%lc of the dose within 120 minutes as indicated in Fig. 3.

#### Dosage uniformity

Dosage uniformity is measured to ensure a constant dose of drug between individual dosage forms. All ALB and TNZ samples were in line with pharmacopoeial acceptance criteria for dosage uniformity, but 19.1% (8/42) of MEB samples did not meet these specifications as indicated in Table 5.

#### Friability test

A relatively high failure rate (20%) of ALB samples followed by TNZ (7.7%) and MEB (7.1%) was observed in the present study. Overall 10.4% (11/106) of samples failed to meet the friability test (S1–2 Supporting information). The higher friability for ALB products might be related to the rapid disintegration and dissolution of these products.

### Risk analysis

The results of the RPN values after scores assigned for severity, occurrence and detectability of the failure mode are presented in Table 7. In the quality attributes subjected to FMEA, a total of 5 failure modes with RPN scores ranging from 2 to 512 were identified. Risk analysis showed that assay (RPN = 512) is the most critical quality attribute followed by dissolution (RPN = 336) and dosage uniformity (RPN 144). Friability was found to be the quality attribute of the least concern according to FMEA analysis applied to product quality assessment.

**Table 7:** Failure mode effects analysis (FMEA) for the different MEB, ALB and TNZ drug product quality attributes.

*#*	*Critical quality attributes (CQA)*	*Failure mode*	*Failure effects*	*Severity*	*Occurrence*	*Detectability*	*Risk priority number (RPN)*
1	Identity	No (intended) active ingredient in the sample or mislabeling (incorrect, inadequate or incomplete identification)	Treatment failure, death due to untreated disease	10	1	5	50
2	Assay	Under-dose, over-dose	Treatment failure, toxicity due to over-dose, drug resistance due to underdose	8	8	8	512
3	Disintegration	Inability to sufficiently disintegrate within the specified time period	No or poor absorption and bioavailability thus leading to treatment failure and resistance	6	1	5	30
4	Dissolution	Inability to sufficiently dissolute within the specified time period	Poor absorption and bioavailability thus leading to treatment failure and resistance	7	6	8	336
4	Dosage uniformity	Non-uniform distribution of dose/content within the individual dosage units	Sub-optimal therapy for a patient taking the sub-standard dosage unit only once and resistance	6	6	4	144
5	Friability	Tablets weight loss due to distribution or any other logistic related factors	Sub-optimal therapy due to loss of the active ingredient	2	1	1	2

### Derringer's desirability function

**Figure 4:**
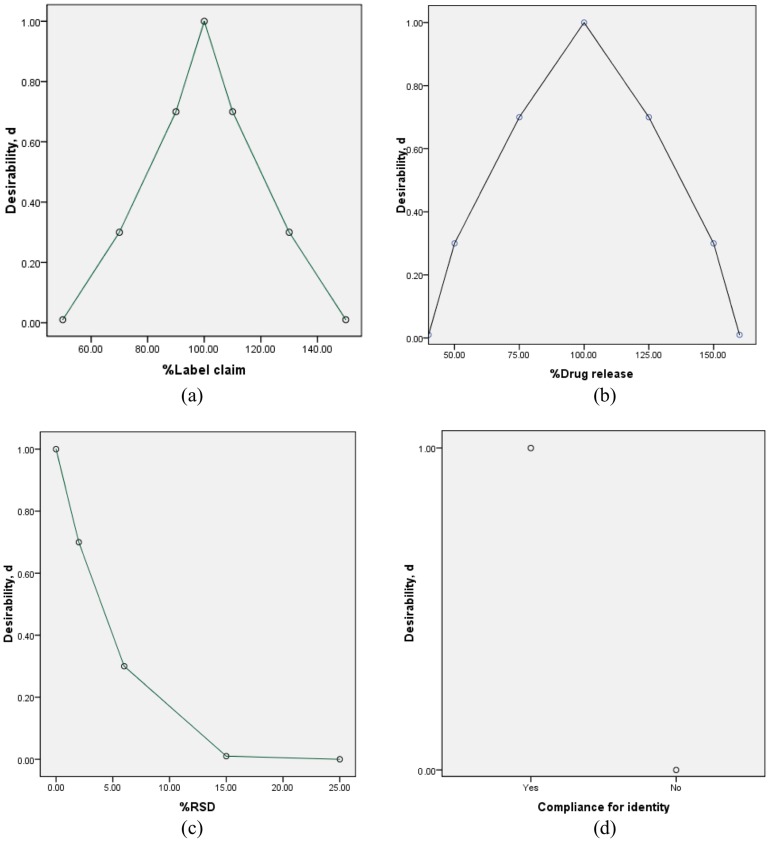
Linear desirability functions: (a) assay (%label claim), (b) dissolution (%drug release), (c) dosage uniformity (%RSD) and (d) identity (compliance to specification).

The results of individual desirability values *d_i_* and the overall desirability D are presented in the S2 supporting information. The individual desirability values assigned to the different segments were fitted to the segmented linear model as indicated in Fig. 4.

**Figure 5:**
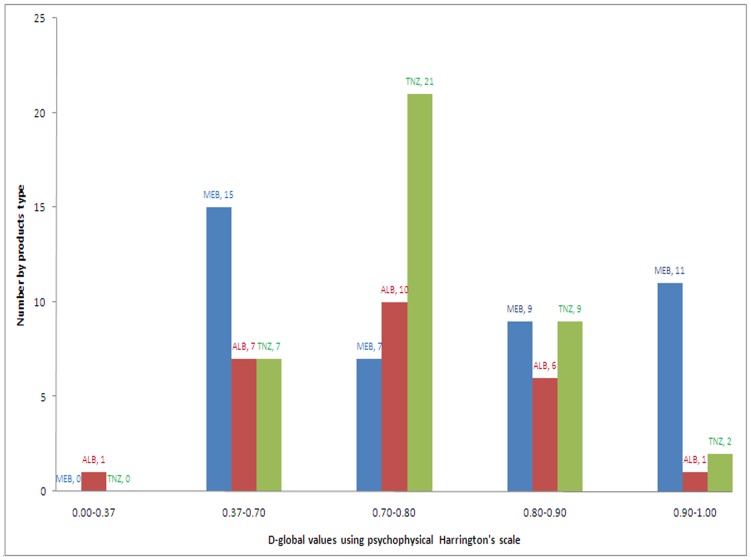
Distribution of D-values among the investigated products.

For each medicine analyzed for the retained 4 quality attributes (assay, dissolution, dosage form uniformity and identity), a global D was finally calculated using the above mentioned d-functions and evaluated using the psychophysical Harrington's scale of quality as presented in Table 4. According to this scale, it was revealed that 13.2% (14/106) of the products were excellent, while 22.6% (24/106) were good and 35.8% (38/106) were of acceptable quality. Thirty products (28.3%) were found to be of unacceptable quality (low and bad). Moreover, the distribution of the D-values among the investigated products is presented in Fig. 5.

## Discussion

To address the subject of quality of medicines, different internationally accepted semantics and definitions are used. In this study, the semantics “poor quality” was used due to the following reasons:

1. We did not find obvious features which suggest that the investigated samples were counterfeit, falsified and/or unregistered; although we did not explicitly search for counterfeits, falsified and/or unregistered medicines. Therefore, without a detailed investigation involving the manufacturer, distribution-chain and health authorities, it is impractical to rule out this perspective.
2. Poor quality can have good manufacturing practice (GMP) and/or good distribution practice (GDP) reasons, and as such, we do not differentiate explicitly between GMP-substandard (at manufacturer) versus degraded substandard (while being good quality at manufacturer). In this study, it was not practical to easily differentiate if poor quality was due to weak GMP or rather inadequate GDP. Moreover, the WHO as well as US-IOM (institute of medicine), and recent expert opinions [39] do not make this distinction and prefer to use the term ‘substandard’ to address both. Finally, quality does also include the intrinsic stability of the medicine (a very important aspect in tropical climates), which is a function of its composition, quality of ingredients/packaging and manufacturing process [40].
3. Even though the term ‘substandard’ literally means “under the standard”, it is obviously related to a legally required specification mostly interpreted in the national regulations in the approved marketing registration file and/or national pharmacopoeia/compendia, where some still use other quality standards, e.g. Ph.Int. or USP-MC. They all differ not only in quality attributes and methods, but also in acceptance criteria as well. As in Low and Middle Income Countries (LMIC), the legally required standards are often absent and incomplete. Therefore, we prefer to use “poor quality” over “substandard”, as for the investigated products, we were not aware of the legally binding national Ethiopian quality standards. Moreover, we did not only use more international quality specifications, but also wanted to introduce the Taguchi-concept in our quality-evaluation, thus (to some extent) avoiding the “standard” issue, with an on-off decision, and replacing it by a quantitative quality number.
4. While other semantics like (S)SFFC, fake, and the like are sometimes used, we believe in accordance with EMA, US-IOM, and recent expert opinions, that for the purpose of this study, a simple 2-dimensional division between falsified and substandard will be sufficient.

In this study, we conducted quality evaluations based on two different approaches: the conventional viewpoint (dichotomous decision based on arbitrarily pharmacopoeial acceptance limits) and the risk-based desirability function approach. The conventional perspective is based on the acceptance criteria set in general and individual monographs of different pharmacopoeias and guidelines, while the desirability function approach is based on quality-by-design (QbD) and risk-based principles whereby clinical relevance is a key factor. A medicine can have many different quality attributes, which are certainly not equally important, i.e. each quality attribute has a different criticality for the clinical use of the medicine. This ICH-recommended risk-based approach is derived from the Taguchi quality philosophy, where any deviation from the optimal point is considered as a less optimal situation and there is no dichotomous decision (see S1-4 Supporting Information).

Therefore, this study reports not only the percentage compliant with the generally accepted pharmacopoeial specification limits for each of the quality attributes using the conventional, dichotomous approach, but also derived a global quality number which encompasses the clinical importance of the different quality attributes. This clinical importance, i.e. criticality or risk if deviating from the optimum, was assessed by quality risk tools: within FMEA, one uses the risk priority number (RPN) to estimate this risk.

### Conventional quality of investigated medicines

In general, the prevalence of poor quality medicines was the highest for ALB tablets (48.0%, 95% CI: 28.4 to 67.6), followed by MEB (45.2%, 95% CI: 30.2 to 60.3) and TNZ (43.6%, 95% CI: 28.0 to 59.2) tablets (Table 5). Overall, 45% (48/106) of the analyzed drug samples failed to meet the official tolerance limits for assay, dissolution, friability and uniformity of dose.

A similar survey conducted on anti-malarial drugs in Senegal, Madagascar and Uganda identified 44%, 30%, and 26% substandard anti-malarial drugs, respectively [41]. Assay and dissolution profile study for anti-malarial samples conducted in south-east Nigeria reported 37% substandard medicines [42]. Assay based pharmaceutical quality assessment in Kenya reported that many anthelmintic preparations marketed in Kenya were of very poor quality [32].

The probable causes for the presence of poor quality medicines in developing countries like Ethiopia might be due to poor storage conditions, insufficient quality assurance, poor compliance with good manufacturing practice standards, lack of scientific expertise in manufacturing sector, limited technical capacity and insufficiently well developed regulatory system to evaluate and take action to solve the problems related to drug quality [43].

From those drug samples collected from pharmacy, about 51.1% (24/46) failed while 46.9% (23/55) and 20.0% (1/5) were the failure rates for those collected from drug store and wholesale, respectively. Even though the sample size was small to generalize, there was significant difference in the pharmacopoeial quality parameter of medicines between the country of origin (P<0.05) but there was no significant association for place of collection and outlets, P>0.05 as presented in Table 8 and Fig. 1.

**Table 8:** Association between the test results and areas of collection, types of drug outlets, and countries of origin.

*Variable*	*Registered quality failure*	*P- value*
**Place of collection (number of samples)**		0.07
Assosa (14)	4/14 (28.6%)	
Hawasa (19)	3/19 (15.8%)	
Addis Ababa (20)	10/20 (50.0%)	
Jimma (16)	10/16 (62.5%)	
Adama (18)	9/18 (50.0%)	
Bahirdar (11)	6/11 (54.5%)	
Mekele (8)	6/8 (75.0%)	
**Total (106)**	**48/106 (45.3%)**	
**Drug outlets (number of samples)**		0.46
Wholesale (5)	1/5 (20.0%)	
Pharmacy (46)	24/46 (52.2%)	
Drug stores (55)	23/55 (41.8%)	
**Total (106)**	**48/106 (45.3%)**	
**Origin (number of samples)**		0.04
Ethiopia (48)	21/48 (43.6%)	
India (28)	11/28 (39.3%)	
Cyrus (15)	8/15 (53.3%)	
China (6)	6/6 (100.0%)	
Korea (9)	2/9 (22.2%)	
** Total (106)**	**48/106 (45.3%)**	

Regarding the collection areas, a high failure rate was observed for samples collected from Addis Ababa, Jimma and Adama areas. Since these areas are commercial centers due to their geographic location, it requires special attention by the regulatory offices to control the circulation of these anthelmintic medicines to combat poor quality medicines circulation.

All analyzed samples contained the intended active ingredient. Even though a single case of API-absent medicine is unacceptable, the finding of this study was good as compared to other studies, e.g. in Cambodia (4.2%) [44]. However, 29.2% (31/106) of the samples did not comply with the pharmacopoeial acceptance criteria for assay. Of the MEB samples, 45.2% were found to be of poor quality with respect to assay as per the official tolerance limit. This result is in agreement with the study conducted in Nigeria's pharmacies in which 48% samples of MEB did not comply with set pharmacopoeial limits [31]. On the other hand, ALB samples showed relatively better compliance but still unacceptable as 8.0% did not meet the official acceptance limit for assay. In general, from those drug samples which failed assay test, 19.4% (6/31) were under-dosed. One of the contributing factors for the development of drug resistance is under-dosing due to poor quality medicines [45].

Uniformity of dosage unit is defined as the degree of uniformity in the amount of active substance among individual dosage units. Content uniformity depends on a number of formulations and manufacturing processes, hence it is obviously unrealistic to presume that every unit contains exactly the same amount of the active ingredient as indicated on the label. Therefore, pharmacopoeial standards and specifications have been established to provide generic limits for allowable variations for the active ingredients in single dosage units considering fitness-for-use and production capability considerations [46]. It was previously reported that (single dose) ALB is more efficacious against hookworm than (triple dose) MEB [47], which may partly be explained by our quality results revealing that all ALB and TNZ samples fulfill the acceptance criteria for dosage uniformity while 19.1% (8/42) of MEB samples did not meet these pharmacopoeial acceptance criteria.

Friability test is conducted to check whether the weight loss during handling is within 1.0% loss specification limit. As indicated in Table 5, 5 ALB, and 3 MEB and 3 TNZ tablet samples failed the pharmacopoeial acceptance criteria of friability. The percent weight loss for all the drug samples failing the specification criteria ranges between 2.2 to 6.0%, where the largest weight loss was registered from a MEB tablet sample. Taking into consideration the single dose regimen and the already substandard drugs with content less than 90%lc, this maximum weight loss from friability study by MEB sample could further pose more risk of drug resistance leading to treatment failure than the other two drugs, ALB and TNZ.

In the present study, since all the drug samples tested for disintegration have met the pharmacopoeial acceptance criteria, there is no risk associated with disintegration as a quality attribute. However, 42% of the ALB samples and 18% of the TNZ samples which were tested for dissolution have been found to be out of the pharmacopoeial specification limit. For low solubility drugs, raw material and process variables could have impact on clinical safety and efficacy through their effects on dissolution. Therefore, the risk of clinical failure is higher for ALB than TNZ as more delayed dissolution was observed, which could be due to changes in the drug substance particle size, failure to control granulation, and increased level of binder in the formulation [48].

The information available on the effectiveness of various BZs derivatives (e.g. ALB and MEB) is somewhat inconsistent [49], [50]. Thus the observations of different therapeutic outcomes have been to some extent attributed to the different polymorphs with different dissolution rates and anthelmintic activities. Solid-state properties play crucial role in dissolution rate and solubility, especially when different polymorphs are involved affecting the *in-vivo* performance of the drugs [51]–[54]. For example, MEB exists as polymorphs and solvates in the solid state. Of particular importance is the difference in the physicochemical properties of the three known polymorphs A, B, and C. The polymorphic forms of MEB display significant differences in solubility and therapeutic efficacy and form C is preferred clinically due to its optimal bioavailability and reduced toxicity. This is important because polymorph A has no anthelmintic activity alone or when present above 30% in polymorphic mixtures. Literatures indicate that at temperatures typically found in countries located in ICH climatic zones III (hot and dry) and IV (hot and humid) trace amounts of form A in tablets significantly accelerate the transformation of the clinically active polymorph C to form A. This transformation significantly reduces the shelf lives and the dissolution rates of these tablets [55].

ALB also exhibits some polymorphic forms by forming solvated crystals. Each of these crystals, including the un-solvated form, may exhibit all the aspects of polymorphism. However, solid state characterization of ALB indicated that both forms are physically quite stable [51]. A literature report indicated that TNZ also exhibits crystal polymorphism [56].

Regarding the use of ALB or MEB, specific attention should be given to the dose appropriate for infants (12 months and less). Apart from the likelihood of both prevalence and intensity being relatively low in infants in areas where soil-transmitted helminthiasis is endemic, there are questions of efficacy and safety when using an anthelmintic drug in very young children [57]. Some studies reveal that the no observed effect level/no observed adverse effect level (NOEL/NOAEL) for ALB is 7 mg/kg/day and that of MEB was found to be 7.8 and 8.4 mg/kg/day in males and females, respectively in experimental animals [58]. Taking the studied ALB tablets, it is possible to assess the associated risk due to the overdosed assay values. The standard treatment guideline for Ethiopia recommends 400 mg tablet as a single dose for treatment of different helminths infections [58]. Assuming an average body weight of 70 kg (body mass index: 23 and height: 175 cm), the NOEL/NOAEL value for ALB can be calculated to be 490 mg per day (taking a safety factor of 1), equivalent with 122.5%lc for a 400 mg tablet. All the assay values for ALB drug products were found to be less than or equal to 111.0%lc, indicating absence of clinically significant risk for the ALB overdosed formulation related to adverse effects. For MEB, since the treatment guideline recommends 200 mg per day [59] and the NOEL/NOAEL value is much higher, the over-dose in the assay values is not a direct clinical concern related to adverse effects.

The assay distribution of the analyzed TNZ samples was found to be from 86.1 to 120.6%lc. Considering the 2 g single dose regimen of TNZ for treatment of giardiasis and the high level NOEL/NOAEL value of 150 mg/kg together with the relative clinical safety of TNZ, the over-dose in the assay values is also not a direct clinical concern related to the adverse effects.

Under-dosing, which could be caused by degradation due to inappropriate storage conditions, might pose toxicity risks due to the degradant impurities. It can be one of the risk factors for the development of anthelmintic resistance. Sub-optimal regimens are the rule in human treatment: anthelmintics are administered in single doses that never achieve 100% efficacy. Taking into account the limited efficacy of single dose anthelmintic treatments, the currently recommended regimens could constitute a significant contributing factor to the development of anthelmintic resistance in STH [60]. In addition to the single dose regimen, the substandard drugs with content less than 90%lc, could further exacerbate the problem of drug resistance leading to treatment failure. Therefore, the risk of development of drug resistance to MEB is higher than the other two drugs, ALB and TNZ since four of the six under-dosed substandard drug samples were MEB.

### Risk-based approach to medicines quality

FMEA is a well-known assessment tool used to identify the critical components most likely to cause failures and to enhance system reliability, through the development of suitable corrective and preventive actions (CAPAs) [61]. Typically, the criticality is evaluated either with the criticality number (CN), or with the risk priority number (RPN). Although the CN is considered more consistent and accurate, the RPN approach is generally preferred, especially for its easiness of use [62], where the higher RPN values indicate the criticality of the quality attribute.

### The desirability function and its application in evaluation of quality of medicines

Optimizing parameters is a critical issue during the development of any method and/or product. A special set of functions called desirability functions have been used in optimizing methods [63], [64] and products characteristics [65], [66]; but the application of such desirability functions for the assessment of the quality of pharmaceutical products is new.

The overall desirability function D is obtained from the individual desirabilities (d_i_) using Equation 2. It can provide a way to assess the quality according to one property, the overall D-value. By mapping all properties onto a desirability scale between 0 and 1, the individual desirability scores due to multiple properties may be easily combined as a geometric mean even if the properties have different scales or units of measurement [67].

In the calculation of the overall D-value using Equation 2, *p_i_* = 3 was used for assay since quality risk associated to it was found to be more important (RPN = 512). Similarly, *p_i_* = 2 was used for dissolution since the risk associated with dissolution was of more concern (RPN = 336) than others. For each of identity and dosage uniformity, *p_i_* = 1 was assigned. The risk assessment revealed that friability was not critically important with calculated RPN value of only 2 and thus was not considered for the desirability study.

The risk analysis conducted indicated that the failure effects due to the failure modes (non-complying quality attributes) was found to be almost similar for the three products analyzed. For example, for all, the over-dose in the assay values was evaluated to be not a direct clinical concern related to the adverse effects. Moreover, since all the three drugs are in BCS class II [26], dissolution is equally a concern. Therefore, the same Derringer's desirability function was applied to all the drug products.

In general, comparing the two quality evaluation approaches, it is reported that 29.2% of the samples were of poor quality when using the pharmacopoeial method of quality evaluation, while it is 28.3% using the new innovative risk-based desirability function approach. Even though it seems that there is no discrepancy between the results of the conventional and D-function approach, we still want to argue that the D-approach provides more weight to the clinically more critical quality attributes and thus fit-for-purpose in resource-limited economies. Resources could thus be prioritized and reliable decisions can be made on the available data using only the clinically more critical quality attributes (assay and dissolution) than the less critical ones (friability and disintegration tests). Moreover, the new QbD and risk-based approach will less heavily penalize marginal out-of-specification medicines, and therefore, we believe it is especially important for poor-resource countries.

In conclusion, this study indicated that all sampled products (MEB, ALB and TNZ) did contain the stated active ingredient, but poor quality products were identified in all three medicines and collection sites in the country due to non-compliant assays, inadequate drug release of required dose or toxicity concerns due to over-dosage of some of the medicines containing higher level of active ingredient. Over-dose in the assay values of the three studied drugs is not a direct clinical concern related to adverse effects where as under-dosing constituted one of the risk factors for the development of resistance.

The study further identified the most critical quality attributes in product quality assessment using FMEA risk-based quality evaluation of the three drugs where assay was found to be the most critical quality attribute with highest RPN. Moreover, it was revealed that Derringer's desirability function can be applied to pharmaceutical quality assessment using Psychophysical Harrington's scale of quality where products could be classified into excellent, good, acceptable, low and bad quality.

Our study suggests policy strategies of containing the problems related to poor quality medicines using this proactive risk-based and desirability function approaches in nation-wide surveillance of the quality of medicines circulating in their respective markets. Furthermore, other possible strategies for containing the problem of these poor quality medicines are e.g.:

- strengthening the capacity of drug regulatory authorities for quality assurance and quality control activities;
- harmonization and regional sharing of information about manufacturing and distribution quality;
- enforcement of regulations and legal prosecutions;
- empowerment and capacity building of medicines inspectors;
- continuous inspection and monitoring of the different levels of medicines supply chain;
- continuous and sustainable product quality surveillance studies with strong monitoring and evaluation activities

## Supporting Information

Supporting Information S1 **The chemical structures of ALB, MEB and TNZ (S1-1); Quality attributes and corresponding pharmacopoeial specifications for ALB, MEB and TNZ tablet products (S1-2); Details of laboratory test methods (S1-3); Experts selected from Belgium and Ethiopia for severity ranking (S1-4); and Risk analysis (S1-5).**
(DOC)

Supporting Information S2 **Sample information together with the experimental results, and individual and global desirability values for each of the investigated medicines.**
(XLS)
