# Supplementary material for: Quality of Medicines Commonly Used in the Treatment of Soil Transmitted Helminths and Giardia in Ethiopia: A Nationwide Survey
Source: PLoS Negl Trop Dis. 2014 Dec 4;8(12):e3345. doi: 10.1371/journal.pntd.0003345 (PMC4256469; doi:10.1371/journal.pntd.0003345)
Supplement: Supporting Information S1 — The chemical structures of ALB, MEB and TNZ (S1-1); Quality attributes and corresponding pharmacopoeial specifications for ALB, MEB and TNZ tablet products (S1-2); Details of laboratory test methods (S1-3); Experts selected from Belgium and Ethiopia for severity ranking (S1-4); and Risk analysis (S1-5). (DOC) [file pntd.0003345.s001.doc]

**Supporting information S1**

**Supporting information S1-1: Chemical structure of albendazole (ALB), mebendazole (MEB) and tinidazole (TNZ)**

**
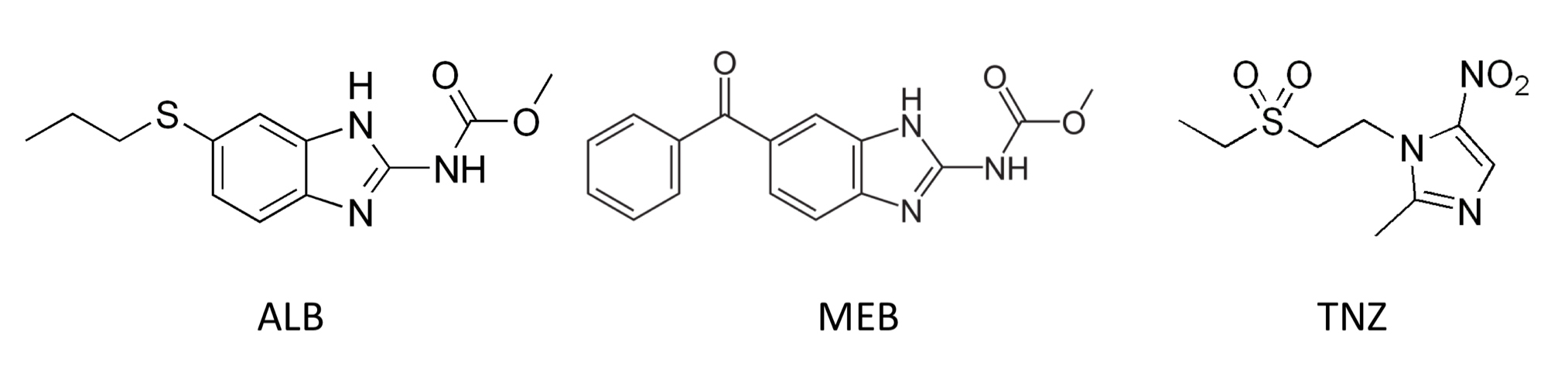
**

**Supporting information S1-2: Quality attributes and corresponding pharmacopoeial specifications for ALB, MEB and TNZ tablet products**

| ***#*** | ***Attributes*** | ***Product*** | ***Specifications*** | | | ***Remarks*** |
| --- | --- | --- | --- | --- | --- | --- |
|  |  |  | ***USP*** | ***Ph Eur*** | ***BP*** |  |
| 1 | Assay (%lc) | ALB, MEB, TNZ | 90.0-110.0 | NA | NA | No individual monographs in BP and Ph Eur |
| 2 | Disintegration time (min) | ALB, TNZ | 15 | 15 | 15 | The specification is based on general monographs |
| 3 | Dissolution (% dissolved within specified time, min) | ALB | Not <80% in 30 min | For the first 6 tablets, no unit not < Q+5 | Not < 70 | The specifications are based on individual monographs for USP |
|  |  | TNZ | Not <75% in 120 min |  | Not < 70 |  |
| 4 | Dosage uniformity (%lc) | ALB, MEB, TNZ | 85.0-115.0 | 85.0-115.0 | 85.0-115.0 | The specification is based on general monographs |
| 5 | Friability (% loss of mass) | ALB, MEB, TNZ | <1 | < 1 | < 1 |  |

NA: Not applicable. Q: the specified amount of dissolved active substance expressed as %lc.

**Supporting information S1-3: Details of the laboratory test methods for products quality**

***Identification test***

Thin layer chromatography (TLC) was used to determine the presence of ALB and MEB, whereas TNZ was identified using UV-spectrophotometry (CECIL CE7200, England) based on the measurement of specific absorbance at 310 nm (USP 2007).

***Assay***

Assay for all the three FPPs was performed using individual monographs in USP 2007. For assay evaluation of MEB and ALB, reversed phase (RP) HPLC system (KONIK HPLC 550A, Model 600, Spain) with LC software coupled to UV-VIS detector was used. The separation technique was carried out using C18, 4.6 mm × 150 mm analytical column that contains 5-µm packing. The HPLC conditions were detection wavelength of 254 nm (Konik 600 UV-VIS detector), isocratic flow rate of 1 ml/min and mobile phase consisting of methanol and 10 mM monobasic ammonium phosphate buffer of pH 5.5 (60:40). Before conducting the assays, the HPLC method used was validated for linearity, precision and accuracy.

ALB sample solutions were prepared from 20 tablets which were weighed and finely powdered. A portion of the powder equivalent to 100 mg of ALB was transferred to a 50.0 ml volumetric flask. Then, 5.0 ml of sulfuric acid in methanol and 25ml of methanol were added and shaken. The solution was diluted and mixed with methanol to volume. 5.0 ml of a clear filtrate (Whatman No. 1 filter paper) of the resulting solution was transferred to a 50.0 ml volumetric flask and diluted to volume using methanol. The reference standard solution was prepared using the same procedure as the sample preparation and both were analyzed using HPLC.

Similarly, MEB sample solutions were prepared from 20 tablets which were weighed and finely powdered. A portion of the powder equivalent to about 500 mg of MEB was transferred to a 100.0 ml volumetric flask. Fifty ml of formic acid was added and heated in a water bath at 50°C for 15 min. The solution was diluted with water to volume and it was mechanically shaken for 1 hour. 5.0 ml of a clear filtrate (Whatman No. 1 filter paper) of the resulting solution was transferred to a 25.0 ml volumetric flask and diluted to volume with the mobile phase. Finally, the diluted solution was mixed and filtered with a sintered glass filter (Millipore®, Darmstadt, Germany) with a pore size of 47 µm. The reference standard solution was prepared using the same procedure as the sample preparation, except the amount of MEB reference standard transferred was 25.0 mg and 10 ml of formic acid was added to the 100.0 ml volumetric flask. Finally, both the sample solutions and the reference were analyzed using HPLC.

For the assay of TNZ, three techniques are available from the literature, giving reliable results of high accuracy and precision: potentiometry, spectrophotometry and HPLC [Basavaiah K, et al., 2005]. However, since USP 2007 uses the potentiometric method, TNZ was assayed using this pharmacopoeial method. Twenty tablets were weighed, finely powdered and a portion of the powder equivalent to 150 mg of TNZ was accurately weighed and dissolved in 25 ml of glacial acetic acid and titrated with 0.1 N perchloric acid standardized volumetric solution. The endpoint was determined potentiometrically using combined glass pH electrode (A1131B, Adwa Instruments, Szeged, Hungary).

Measured drug content was expressed as a percent of labeled claim (%lc). According to USP individual monographs for ALB and MEB tablets, they should contain not less than 90% and not more than 110% of the labeled amount (USP 2007). Since there was no specific assay specification for TNZ tablets in USP, the same acceptance limits as used for ALB and MEB tablets were applied.

***Disintegration test***

With the assumption that the patient swallows with proper chewing, the disintegration test was not performed for MEB chewable tablets.

Disintegration test was thus carried out for ALB and TNZ tablet samples using USP <701> general monograph. Six tablets from each drug product were tested for disintegration times in 900 ml distilled water at 37±2 ºC using a disintegration apparatus A (PTZ-2E, PharmaTest, Germany) without disk. The disintegration time was taken to be the time at which no tablet was left on the meshes of the apparatus under given set of conditions.

The general USP monograph for tablets requires that uncoated tablets, except for chewable tablets which are exempted from this test, disintegrate within 15 min.

***Dissolution study***

An *in-vitro* dissolution study was carried out for TNZ and ALB using a six vessels Dissolution Tester (Tian-Jin, RC-6D, China) following individual monographs in USP 2007. Dissolution study was not conducted for MEB samples since there were no sufficient samples left from the other tests.

For ALB tablets, it was performed using USP type II dissolution apparatus (paddle), while for TNZ apparatus type I (basket) was used. The dissolution medium was 900 ml of 0.1N hydrochloric acid at a temperature of 37±1^o^C and 50 rpm. Dissolution was carried out based on the pharmacopoeial defined timings for complete dissolution, which was 30 minutes for ALB and 120 minutes for TNZ. Considering the rapid disintegration and dissolution, 4 ALB products (3 brands and 1 generic product) and 7 TNZ products (4 brands and 3 generic products) were purposefully selected to represent the products and tested for a complete release profile. At time intervals of 5 min for ALB and 10 min for TNZ, samples of 10.0 ml were withdrawn and replenished with an equal volume of fresh dissolution medium at the same temperature. Samples were filtered using Whatman No. 1 filter paper, suitably diluted and assayed using UV/Visible spectrophotometer (CECIL CE7200, England) at 350 nm and 317 nm, respectively. The calibration curves for ALB and TNZ were prepared as specified in individual monographs in USP 2007 by preparing six different concentrations from stock solution. The percent cumulative release was calculated and plotted against time of release.

For ALB tablets, the official tolerance limits according to USP individual monograph is that more than 80.0% should be released within 30 min, where as for TNZ tablets, more than 75.0% of the dose should be released within 120 min.

***Dosage uniformity***

The dose uniformity of tablets can be determined by two general approaches: the weight variation between the sampled tablets or the drug content uniformity. In this study, the weight variation test was performed as a means of quantifying uniformity of dose units since all tablet samples contain 100 mg or more active ingredient, i.e. all the drug products have a dose greater than 25 mg and a ratio of drug substance over finished product greater than 25% (USP 2007, Ph. Eur 2012). Ten tablets were selected at random, weighed all together using the analytical balance (Mettler Toledo, AL-204 AC, USA) and the average weight was calculated. Then, each tablet was weighed individually and the percentage deviation from average weight was calculated. The acceptance value was calculated using the individual tablet weights and the assay result. The requirements for dosage uniformity are met if the acceptance value of 10 dosage unit is less than or equal to L1% (15.0). If the acceptance value is greater than L1%, further 20 tablets were taken and calculated for the acceptance value, which should then be below L2% (25.0).

***Friability test***

To evaluate the friability of the tablets of each drug product, a number of tablets, adding up to 6.5 g, were taken for MEB (unit mass < 650 mg), while 10 tablets were taken for ALB and TNZ (unit mass > 650 mg) (USP 2007, Ph. Eur. 2012). Prior to the test, the tablets were carefully de-dusted and accurately weighed. The tablets were then placed in the drum of the friability test apparatus, PharmaTest Friabilator (PTF-20E, PharmaTest, Germany) and subjected to its tumbling action at 25 rpm for 4 min. After 4 minutes only intact tablets were once again de-dusted and weighed to determine the percentage weight loss by tablets due to mechanical action during test. If cracked, cleaved, or broken tablets were present in the tablet sample after tumbling, the sample was considered to fail the friability test.

According to USP general monograph, a maximum mean weight loss of not more than 1.0% is considered acceptable for most products (USP 2007).

**Supporting information S1-4: Experts selected from Belgium and Ethiopia for severity ranking**

For severity ratings, five pharmaceutical experts in Belgium (4) and Ethiopia (1) were assigned. They were selected based on their level and area of qualifications. The level of qualifications is pharmacists (finalizing PhD), PhD and Professors in the area of pharmaceutical quality, analytics and regulatory sciences.

**Supporting information S1-5: Risk analysis**

Risk analysis is a general quality tool which indeed has its roots in engineering (eg Harry et al Wiley 2010: Practitioner’s guide to statistics and lean six sigma for process improvements) and environmental sciences (eg REACH in EU), but is now becoming a well-established tool in the pharmaceutical field as well. As such, ICH has devoted a separate guideline (Q9) to quality risk management, which is being embraced by pharmaceutical authorities (ICH Q9, 2006). Risk analysis, i.e. the estimation of the risk associated with the identified hazards, is an important part of this global risk management. Several quality risk management methods and tools are available, as mentioned by ICH in Q9, e.g. FMEA (Failure Mode Effects Analysis). FMEA is thus a tool to assess the risks from potential product failure. And here is one of our innovations:

1) while a medicine has many different quality attributes, they are certainly not equally “important”, i.e. each quality attribute has a different criticality for the clinical use of the medicine (clinical relevance is key in aQbD of medicines).

2) while the traditional view is a dichotomous approach (product is considered equally good if within the acceptance or specification limits, e.g. 91% and 100% label claim for the assay are considered equal quality, and suddenly unacceptable if outside these limits, e.g. 89% label claim if underlimit was 90%), the ICH-recommended risk-based approach is derived from the Taguchi quality philosophy, where any deviation from the optimal point is considered as a less optimal situation and there is no dichotomous decision.


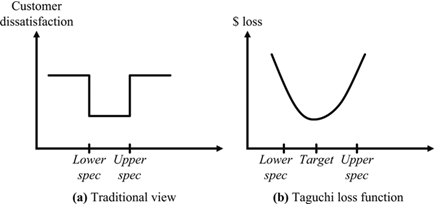


In our manuscript, we have applied both approaches: not only the percentage compliant with generally accepted specification limits for each of the quality attributes (the traditional, dichotomous approach), but we have also derived a global quality number which encompasses the clinical importance of the different quality attributes. This clinical importance, i.e. criticality or risk if deviating from the optimum, was assessed by quality risk tools: within FMEA, one uses the risk probability number (RPN) to estimate this risk. As this RPN-concept is relatively new in the pharmaceutical field, we have included Tables 1 to 3 in our manuscript for explanatory reasons. The same is true with the desirability concept, which combines different quality attributes into one global quality figure (Table 5 in the manuscript was given for explanatory reasons).

In a resource limited settings, we believe that such type of risk-based quality evaluation is very important, as such an approach is more focused towards the clinically important quality attributes. This study reported that assay was found to be the most critical quality attribute followed by dissolution, whereas friability was the least critical one.
